# Thyrotoxic Periodic Paralysis: The Peril of Potassium Replacement

**DOI:** 10.7759/cureus.32926

**Published:** 2022-12-25

**Authors:** Sean Ho Yoon, Hassan Raza

**Affiliations:** 1 Internal Medicine, NewYork-Presbyterian Queens, Flushing, USA; 2 Internal Medicine, NewYork-Presbyterian Queens, Queens, USA

**Keywords:** internal medicine, hypokalemia, periodic paralysis, thyrotoxicosis, endocrinology

## Abstract

Hypokalemic periodic paralysis (HPP) is a clinical condition of sudden-onset, recurrent transient episodes of weakness caused by severe hypokalemia. Thyrotoxic periodic paralysis (TPP) is a specific subgroup of the HPP spectrum, where hypokalemia occurs in the setting of thyrotoxicosis, and the repletion of potassium must be performed with caution.

A male in his second decade of life with hyperthyroidism non-adherent to methimazole presented with acute-onset bilateral lower extremity weakness. On physical examination, the patient had diffuse thyromegaly and tremors on outstretched hands. Bilateral lower extremity weakness and decreased reflexes were also noted, with preserved muscle tone and normal passive range of motion. Labs demonstrated hyperactive thyroid function and severe hypokalemia at 1.7 mEq/L, with U waves present on the electrocardiogram. In the intensive care unit, the patient received methimazole and propranolol for thyrotoxicosis and a total dose of 60 mEq/L of potassium replacement therapy. Despite the expected correction by 0.6 mEq/L, his follow-up potassium level dramatically increased to 5.7 mEq/L, resulting in the actual correction of 4.0 mEq/L. Within a few hours, the patient regained his baseline strength with a significant improvement in tremors.

Patients with TPP present with acute-onset extremity weakness and severe hypokalemia, which reverses quickly with potassium repletion. Clinicians should not only treat thyrotoxicosis but also avoid overly aggressive repletion of potassium as this may lead to rebound hyperkalemia when the initial transcellular potassium shift is reversed.

## Introduction

Hypokalemic periodic paralysis (HPP) is a clinical condition of sudden-onset, recurrent transient episodes of generalized muscle weakness caused by severe hypokalemia, defined as a serum potassium level less than 2.5 mEq/L [1]. Severe hypokalemia is a life-threatening electrolyte imbalance that may lead to fatal cardiac arrhythmia and death. For patients who are diagnosed with HPP, it is generally recommended to avidly replete missing potassium [2]. However, overzealous correction of hypokalemia may also lead to rebound hyperkalemia (>5.0 mEq/L), which is likewise a potentially fatal condition that can lead to cardiac arrhythmia and eventual respiratory arrest. We present a case of thyrotoxic periodic paralysis (TPP), a specific subgroup of the HPP spectrum, where hypokalemia occurs in the setting of thyrotoxicosis, and the repletion of potassium must be performed with caution.

## Case presentation

**Figure 1:**
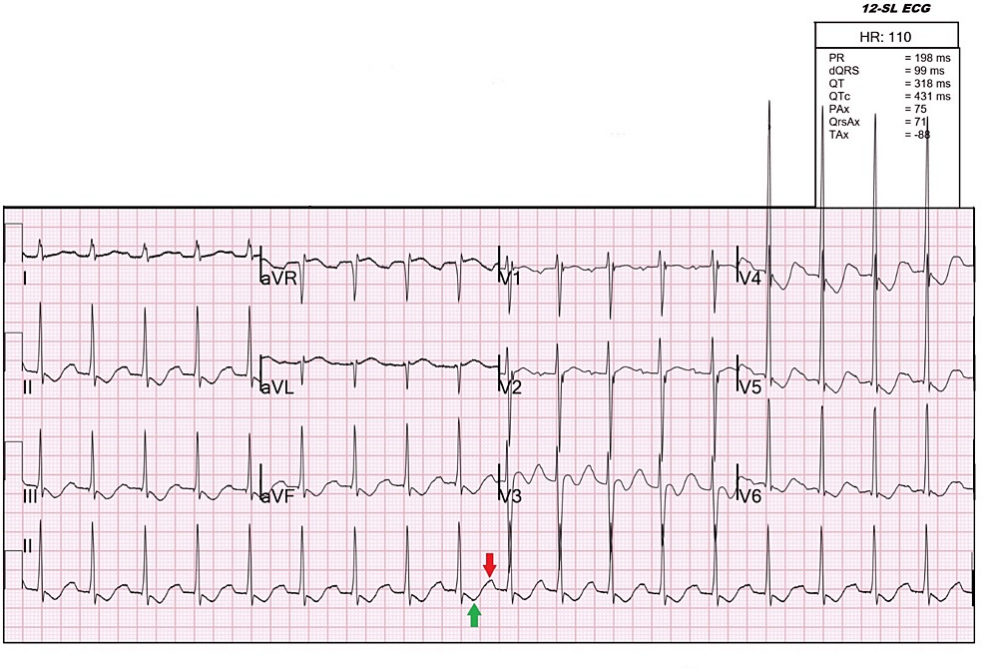
The electrocardiogram of the patient in the setting of thyrotoxic periodic paralysis with hypokalemia at 1.7 mEq/L. Note the sinus tachycardia with a heart rate of 110 beats per minute, inverted T wave (green arrow), and prominent U wave (red arrow).

A male in his second decade of life with hyperthyroidism diagnosed in 2017 non-adherent to methimazole presented with acute-onset bilateral lower extremity weakness. At symptom onset, the patient was unable to flex both lower extremities when he woke up in the morning. The patient stated he had a similar episode of extremity weakness a few years ago, where he was incidentally found to be hypokalemic. The patient was recently involved in strenuous physical activity leading to lower extremity muscle soreness, followed by palpitations, tremulousness, and episodic watery diarrhea over the past two weeks. In the emergency department (ED), vital signs were only remarkable for sinus tachycardia to 110 beats per minute. On physical examination, the patient had diffuse thyromegaly and tremors on outstretched hands. Bilateral lower extremity weakness (Medical Research Council (MRC) scale of power 1/5 in both lower extremities) and decreased reflexes were also noted, with otherwise preserved muscle tone and normal passive range of motion. Labs were significant for severe hypokalemia at 1.7 mEq/L (3.6-5.2 mEq/L), with U waves present on electrocardiogram (ECG) (Figure 1), thyroid-stimulating hormone less than 0.010 mU/L (0.45-4.5 mU/L), elevated total T3 of 335.7 ng/dL (60-180 ng/dL), with free T4 of 6.24 ng/dL (0.6-1.3 ng/dL). Other electrolytes showed magnesium of 2.1 mEq/L (1.3-2.1 mEq/L) and phosphorus of 3.3 mEq/L (2.8-4.5 mEq/L). Otherwise, the creatine kinase level was normal, and no acid-base disturbance was found.

**Figure 2:**
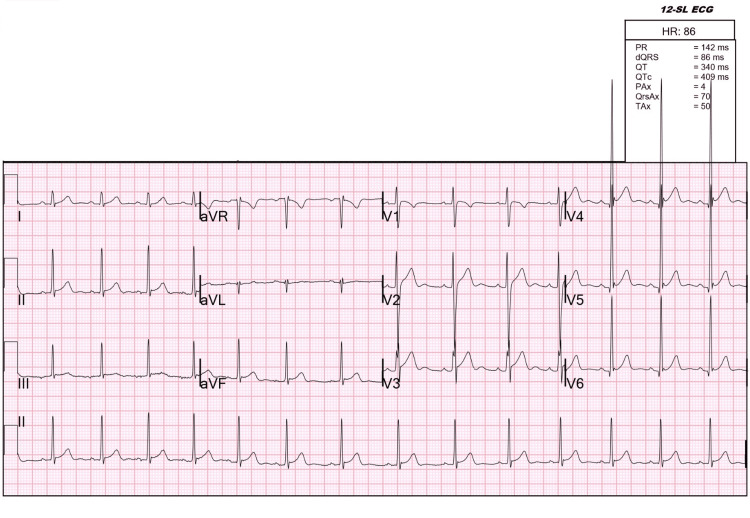
The electrocardiogram following the treatment of thyrotoxic periodic paralysis. Note the near resolution of U waves and normal sinus rhythm. The U waves still seen in leads V2, V3, V4, and V5.

In the setting of profound acute lower extremity weakness and the degree of thyrotoxicosis, the patient required vital sign measurements and neurological assessment every hour. In the intensive care unit, the patient received methimazole oral route 20 mg every 12 hours and propranolol oral route 40 mg every eight hours to treat thyrotoxicosis and a total dose of 60 mEq/L of potassium replacement therapy over six hours. After replacement, despite the expected correction by 0.6 mEq/L, his follow-up potassium level dramatically increased to 5.7 mEq/L, resulting in the actual correction of 4.0 mEq/L. Within hours of initiation of therapy requiring a relatively small dose of potassium supplement, the patient regained his baseline strength in his extremities along with a significant improvement in tremors. Repeat ECG showed normal sinus rhythm with near resolution of U waves that were initially seen (Figure 2). The patient was then discharged with a close endocrinology follow-up for his thyroid condition.

## Discussion

As seen in our case, it is crucial to identify TPP in a patient presenting with acute-onset extremity weakness, ranging from mild weakness to complete flaccid paralysis. In contrast to the hereditary nature of HPP that typically involves skeletal muscle dihydropyridine receptor gene mutations, TPP is a sporadic disease found predominantly in males of Asian ethnicity in the third decade of life. The incidence of TPP is approximately 2% among Asians with thyrotoxicosis of any cause [3,4]. Symptoms usually begin at nighttime but are noticed in the morning, and risk-enhancing behaviors include ingestion of carbohydrate-rich meals or sweets and strenuous physical activity followed by rest, as observed in our case. It is important to note that because hyperthyroidism is not always clinically apparent, obtaining thyroid function tests is generally warranted, especially for those who have known thyroid disease or similar episodes in the past [4,5]. The pathophysiology of TPP involves a state of adrenergic excess, causing Na-K-ATPase hyperactivity with the resultant transcellular shifting of potassium in the setting of normal total body potassium [2]. Non-selective beta-blocker used for thyrotoxicosis further contributes to the rapid reversal of the initial intracellular potassium shift by directly blocking the Na-K-ATPase. Therefore, clinicians should not only treat underlying thyrotoxicosis but also avoid overly aggressive repletion of potassium, as this may lead to rebound hyperkalemia when the initial transcellular potassium shift is reversed as the adrenergic excess state resolves.

In our case, the patient was given a total of 60 mEq/L of potassium, which led to an unmatched increase in potassium level, despite the expected correction by approximately 0.6 mEq/L according to the following potassium deficit formula [6]: Kdeficit (in mmol) = (Knormal lower limit − Kmeasured) × kg body weight × 0.4.

One study demonstrated rebound hyperkalemia (K > 5.0 mEq/L) in approximately 40% of patients with TPP who received potassium of 90 mEq/L or more within 24 hours [7]. To avoid such overcorrection, we advise that repeat serum potassium levels should be obtained after the initial administration of 50 mEq/L or less of potassium replacement. Such practice was shown to substantially reduce the risk of rebound hyperkalemia, demonstrating a dose-driven response [8]. Given the clinical rarity and its application of basic electrolyte physiology in a clinical setting, we feel this case provides an important lesson to clinicians globally.

## Conclusions

Patients with TPP present with acute-onset varying degrees of extremity weakness and severe hypokalemia, which reverses quickly with potassium repletion. Clinicians should not only treat thyrotoxicosis but also avoid overly aggressive repletion of potassium. If not practiced with caution, this may lead to rebound hyperkalemia when the initial transcellular potassium shift is reversed following the resolution of the adrenergic excess state under thyrotoxicosis.
